# Insights into the Psychrophilic and Sea Ice-Specific Lifestyle of *Marinobacter* sp. Strain AC-23: a Genomic Approach

**DOI:** 10.1128/genomeA.00134-17

**Published:** 2017-04-13

**Authors:** Neelam Kapse, Purnima Singh, Utpal Roy, Shiv Mohan Singh, Prashant K. Dhakephalkar

**Affiliations:** a Agharkar Research Institute (ARI), Maharashtra Association for Cultivation of Science, Pune, India; b Birla Institute of Technology and Science (BITS), Pilani-K.K. Birla Goa Campus, Zuarinagar, Goa, India; c National Centre for Antarctic and Ocean Research (NCAOR), Ministry of Earth Sciences, Vasco-Da-Gama, Goa, India

## Abstract

*Marinobacter* sp. strain AC-23 was isolated from Kongsfjorden in the Arctic. Here, we report the first draft genome sequence of a putative novel species of the genus *Marinobacter* comprising 4,149,715 bp, with a mean G+C content of 54.4%. The draft genome sequence will aid in understanding the psychrophilic and sea ice-specific lifestyle.

## GENOME ANNOUNCEMENT

Kongsfjorden is one of the largest glacial fjords of Svalbard (1). Recently, the genome of *Cryobacterium* sp. strain MLB-32 from glacier (2) and the elemental composition of the sediments of fjords (3) have been contributed from Svalbard. AC-23, a Gram-negative, rod shaped, and psychrophilic bacterial isolate, was obtained from 29,370 ± 640-year-old sediments of Kongsfjorden.

Phylogenetic analyses based on 16S rRNA gene sequence revealed *Marinobacter antarcticus* ZS2-30^T^ to be the closest phylogenetic neighbor of AC-23, with 97.87% homology (1,534 nucleotides). Digital DNA-DNA hybridization (4) revealed only 26.5% ± 2.4% homology between AC-23 and *Marinobacter psychrophilus*, indicating the novelty of strain AC-23.

The genome of AC-23 was sequenced with a whole-genome shotgun strategy using 318 Chip and 200-bp chemistry on the Ion Torrent PGM platform (Life Technologies, Inc., USA). A total of 4,149,715 high-quality reads were generated upon sequencing of the genome. *De novo* assembly was performed using MIRA assembler version 4.0.5 (5), resulting in 105 contigs. Functional annotation was performed using the Rapid Annotations using Subsystems Technology (RAST) server (6). The RAST tool identified *Marinobacter aquaeolei* VT8 as the closest phylogenetic neighbor of AC-23 (score, 530; genome identification [ID] 351348.5). The annotation predicted 4,615 genes, including 4,564 coding sequences (CDSs), and 51 total RNAs. The majority of the protein-coding genes (53%) were assigned a putative function, while those remaining were annotated as hypothetical proteins. AC-23 harbors a total of 229 unique genes associated with a subsystem (a set of functional roles that make up a metabolic pathway, a complex, or a class of proteins) when comparative analysis was performed with VT8.

The genome annotation of AC-23 revealed the presence of multiple genes involved in biofilm formation, including 41 homologs of genes encoding pilus IV and four clusters of polysaccharide biosynthesis genes, providing insights into molecular mechanisms of biofilm formation and, more specifically, on strategies of colonization of nutritive surfaces in marine environments. Genetic features that can be linked to the psychrophilic and sea ice-specific lifestyle of AC-23 were analyzed. The major cold shock protein-coding genes, *cspA* and *cspC* (7, 8), and a number of regulatory genes, such as *cpxR*, *algZ*, *cheY*, and *cheC*, that might play a key role in low-temperature adaptability were detected. Modulation of membrane fluidity is a critical adaptation strategy in cold environments (9–11). The AC-23 genome encodes proteins involved in fatty acid biosynthetic pathways, such as FabG, which catalyzes the condensation of fatty acids and the synthesis of branched fatty acids. The genome also codes for three orthologs of 1-acyl-*sn*-glycerol-3-phosphate acyltransferase (PlsC), which catalyzes the conversion of intermediates in phospholipid synthesis, and 3-ketoacyl-(acyl-carrier-protein) reductases, which enhance the production of polyunsaturated lipids (12, 13).

Genome analysis revealed the presence of cold-adapted lipase, protease, d-hydantoinase, catalase, peptidase, etc., with considerable biotechnological potential as cold-active enzymes.

The genome sequence of AC-23 will aid in gaining valuable insights into the cold adaptation of psychrophilic bacteria, and its metabolic potential and will open up new opportunities related to the functional genomics of this species.

### Accession number(s).

The whole-genome shotgun project has been deposited in DDBJ/EMBL/GenBank under accession number MBPP01000000 (BioProject PRJNA329576, Biosample SAMN05415084).
